# Racial and Ethnic Differences in Maternal Health and Autism

**DOI:** 10.1001/jamanetworkopen.2026.37501

**Published:** 2026-10-05

**Authors:** Khaoula Ben Messaoud, Vahe Khachadourian, Meredith Anderson, Magdalena Janecka, Lisa A. Croen

**Affiliations:** 1 Department of Child and Adolescent Psychiatry, New York University Grossman School of Medicine; 2 Division of Research, Kaiser Permanente Northern California, Pleasanton, California; 3 Department of Population Health, New York University Grossman School of Medicine; 4 Kaiser Permanente School of Medicine, Pasadena, California

## Abstract

**Importance:**

Autism affects 1 in every 31 children in the US, with significant racial and ethnic disparities in prevalence, age at diagnosis, and access to services. Maternal health conditions during pregnancy have been linked with autism in offspring. However, the extent to which these associations vary by maternal race and ethnicity remains understudied.

**Objective:**

To evaluate whether associations between maternal conditions during pregnancy and the risk of autism in offspring differ by maternal race and ethnicity.

**Design, Setting, and Participants:**

This retrospective cohort study included children born at Kaiser Permanente Northern California from 2010 to 2017, followed up until February 29, 2020, autism diagnosis, disenrollment, or death. Data were analyzed from September 2024 to July 2026.

**Exposures:**

Maternal *International Classification of Diseases, Ninth Revision* and *Tenth Revision* diagnoses of any of 25 conditions previously identified as associated with autism during the perinatal period.

**Main Outcomes and Measures:**

Cox proportional hazards models examined associations between maternal diagnoses and autism with racial and ethnic differences evaluated via stratified and interaction models. Covariates included maternal age at delivery, insurance type, child sex, birth year, and maternal health care utilization.

**Results:**

Of 224 353 children, 5448 (2.4%) were diagnosed with autism, with prevalence varying by maternal race and ethnicity (493 Black [3.2%], 1693 Asian and Pacific Islander [2.9%], 1309 Hispanic [2.5%], 1838 White [2.0%], and 115 other or unknown race and ethnicity [2.4%] children; percentages indicate prevalence within each race and ethnicity group). Associations between maternal health conditions during pregnancy and offspring autism were largely consistent across racial and ethnic groups. Differences in risk estimates by maternal race and ethnicity were observed for 4 of the 25 conditions, including overweight and obesity, depressive episode, asthma, and other maternal disease complicating pregnancy. All those conditions exhibited stronger associations with autism in White mothers, compared with Black, Hispanic, and Asian and Pacific Islander mothers.

**Conclusions and Relevance:**

In this cohort study of 224 353 children-mother pairs, maternal health conditions during pregnancy showed consistent observational associations with autism across racial and ethnic groups, but some exhibited heterogeneous association estimates. These differences may reflect disparities in health care access, diagnostic practices, or unmeasured environmental and genetic factors. Further research is needed to clarify underlying mechanisms and implications for maternal and child health.

## Key Points

**Question:**

Do associations between maternal health conditions during pregnancy and offspring autism vary by maternal race and ethnicity?

**Findings:**

In this cohort study of 224 353 children, autism prevalence differed by maternal race and ethnicity, but most maternal conditions showed similar association patterns with autism across groups. Four of 25 conditions, including obesity, asthma, and psychiatric disorders, showed racial and ethnic differences in the magnitude of association.

**Meaning:**

Maternal conditions during pregnancy are broadly associated with autism across racial and ethnic groups, but variation in association strength for some conditions suggests that inequities in care or unmeasured factors may contribute to these observed patterns.

## Teaser

This cohort study evaluates whether associations between maternal conditions during pregnancy and the risk of autism in offspring differ by maternal race and ethnicity.

## Introduction

Autism is a developmental condition characterized by differences in social communication and repetitive or restrictive behaviors. The prevalence of autism among children aged 8 years in the US is estimated by the CDC to be 1 in 31 (3.2%), with recent increases in diagnoses, particularly among females and children from racial and ethnic minority groups.^1,2^

A growing body of research has identified associations between several maternal conditions during pregnancy and autism in the offspring, including, but not limited to, obesity, diabetes, hypertensive disorders of pregnancy, asthma, and psychiatric conditions such as depression and anxiety.^3^ We have demonstrated that these associations largely replicate across Danish and Californian settings, despite differences in health care system structures, prevalence of maternal conditions, and racial and ethnic composition of these settings. However, the statistical adjustment for maternal race and ethnicity in our previous study comparing these settings precluded the estimation of the distinct effects of maternal diagnoses among the diverse groups of mothers.^4^

Approaches not specifically addressing heterogeneity in associations across racial and ethnic groups could obscure that the associations between maternal health and autism may be also driven by access to and quality of prenatal care^5^ or by exposure to a range of hazardous neighborhood-level exposures, including air pollution, substandard housing, and limited green space,^6^ which vary across racial and ethnic groups. These differences include residential segregation and resource deprivation, shaping both environmental exposures and access to care. Consistent with this, studies have shown that pregnant individuals from racial and ethnic minority groups often face more obstacles to obtaining health care, receive lower-quality care once they access it, and experience poorer health outcomes.^7,8,9,10^ The disparities in the rates of maternal diagnoses linked with autism across maternal race and ethnicity also suggest potential differences in how these diagnoses are made, or both. Such variation in the correlates of maternal diagnoses suggests that their association with child outcomes may not be uniform across racial and ethnic groups, and merits interrogating their effects in specific population strata.

We utilized a large and diverse cohort of children born within the Kaiser Permanente Northern California (KPNC) health care system to investigate how the association between maternal diagnoses during pregnancy and the estimated likelihood of autism in offspring differs by maternal race and ethnicity. Identifying any disparities in risk across racial and ethnic groups will enable further study into potential causes of such disparities and the development of strategies to improve developmental outcomes of children.^11^

## Methods

### Study Population

The study cohort consisted of children born within the KPNC health care system between January 1, 2010, and December 31, 2017. Children were excluded if they had no KPNC enrollment after birth, if maternal age at delivery was younger than 13 or older than 55 years, or if key covariate data were missing. Mothers were required to be KPNC members for at least 3 months in each of the 2 years prior to delivery.

The follow-up period for each child started at birth and ended at the earliest occurrence of any of the following: February 29, 2020, a first diagnosis of autism spectrum disorder (ASD) after 12 months of age, disenrollment from KPNC, or death. This report follows the Strengthening the Reporting of Observational Studies in Epidemiology (STROBE) reporting guideline for cohort studies. Study procedures were approved by the KPNC institutional review board. The requirement for informed consent was waived because only deidentified administrative data were used.

KPNC is a large, integrated health care system that serves over 4.5 million individuals. The sociodemographic characteristics of its members are broadly representative of California’s general population in the counties served by KPNC, with the exception of underrepresentation among individuals at the extremes of the income distribution.^12,13^

### Exposure

Exposures were the *International Classification of Diseases, Ninth Revision (ICD-9) *and* Tenth Revision (ICD-10)* diagnoses recorded during the perinatal period in the mother, shown to be significantly associated with autism in our previous work, including in the KPNC system.^3,4^ All maternal diagnoses were identified via electronic health records (EHRs) from inpatient and outpatient encounters with a KPNC provider (see eTable 1 in Supplement 1). Maternal diagnoses were not mutually exclusive; a mother could have more than 1 condition, with our previous work finding limited impact of comorbidity on the estimates associated with most diagnoses.^4^ The Chronic Condition Indicator, developed by the Agency for Healthcare Research and Quality,^14^ was used to classify diagnoses into chronic or nonchronic. For nonchronic conditions, we classified children as exposed if their mother received a given diagnosis within 12 months before childbirth. For chronic conditions, a longer detection window of 48 months was used, reflecting the enduring nature of these diagnoses and the difficulty of accurately identifying their onset using EHRs. This broader time frame enhances the sensitivity for capturing maternal chronic conditions that may not be actively managed during pregnancy but may still affect fetal development. Sensitivity analyses exploring the impact of varying detection windows for chronic conditions are reported elsewhere.^3^

### Outcome

The outcome was an ASD diagnosis after the child reached age 12 months, as recorded in the child’s EHR and based on the *Diagnostic and Statistical Manual of Mental Disorders* (Fourth Edition) or *Diagnostic and Statistical Manual of Mental Disorders* (Fifth Edition) criteria. Among children diagnosed with ASD, 85% underwent evaluation at a specialized KPNC ASD assessment center, where standardized procedures, including the Autism Diagnostic Observation Schedule, were used by a multidisciplinary team. The remaining 15% received diagnoses from qualified clinicians such as child psychiatrists, developmental-behavioral pediatricians, pediatric neurologists, or general pediatricians. ASD diagnoses received before age 12 months were not included due to the lack of specificity of diagnosis at such an early age.

### Covariates

Covariates were extracted from the KPNC EHR and included maternal age at delivery, type of insurance at delivery (categorized as KPNC insurance vs government insurance), child’s year of birth, and sex. Insurance type served as a proxy for socioeconomic status (SES). Additionally, the number of days the mother utilized health care services in the year before childbirth was used as a proxy for health care utilization, encompassing both primary and specialist care visits. Race and ethnicity categories were defined in the health system and included Asian and Pacific Islander, Black, Hispanic, White, and other or unknown. The other or unknown category was heterogeneous (see eTable 2 in Supplement 1 for exact composition, derived from the KPNC Virtual Data Warehouse demographic table).

### Statistical Analysis

Analytical procedures were aligned with those described before.^3,15^ Each maternal diagnosis was independently examined for its association with autism using Cox proportional hazards models, with stepwise adjustment for covariates.^16^ To account for the clustering of siblings within families, we calculated robust sandwich estimators. The heterogeneity of associations by race and ethnicity was evaluated by entering an interaction term between maternal diagnosis and maternal race and ethnicity in 1 set of regression models. Because 25 conditions were tested across racial and ethnic strata, we controlled the false discovery rate using the Benjamini-Hochberg procedure. Additionally, we conducted analyses stratified by maternal race and ethnicity to evaluate the association of each diagnosis with autism in each race and ethnicity group. Diagnoses reported in fewer than 5 mothers of children with or without autism in each race and ethnicity group were excluded to reduce the likelihood of sparse data bias.^17^ The only exceptions were diagnoses where the only sparse group were children in the other race or ethnicity category; these diagnoses were included in the analyses, but the individuals whose mothers reported other were excluded from the analytical sample. Diagnoses for which the interaction term was statistically significant (Q value <.05) were considered to have differential associations with autism across racial and ethnic subgroups. Two-sided Wald tests with robust (sandwich) standard errors were used to calculate *P* values for the Cox models, and significance was set at less than .05. Data were analyzed using RStudio software versions 2024.09.0 and 2026.07.1 (R Project for Statistical Computing) from September 2024 to July 2026.

## Results

The study population included 227 195 children. After excluding 1 child (<0.01%) with unknown sex and 2841 children (1.2%) whose insurance type at delivery was missing, the study sample included 224 353 children. The participant flow diagram is detailed in Figure 1. The median (IQR) duration of follow-up was 3.7 (1.3-6.4) years. A total of 5448 children (2.4%) received an ASD diagnosis during the study follow-up period. The median (IQR) age at diagnosis was 3.0 (2.2-4.0) years. The median (IQR) maternal age at the time of delivery was 31.9 (28.3-35.3) years. eTable 3 in Supplement 1 provides the distribution of demographic characteristics and other covariates by maternal race and ethnicity. The prevalence of autism was highest in children born to Black mothers (493 children [3.2%]) followed by Asian and Pacific Islander mothers (1693 children [2.9%]), Hispanic mothers (1309 children [2.5%]), and those of other or unknown race and ethnicity (115 children [2.4%]) and was lowest among White mothers (1838 children [2.0%]) (eTable 3 in Supplement 1). eTable 4 in Supplement 1 presents the demographic characteristics by autism diagnosis and maternal race and ethnicity.

**Figure 1.**
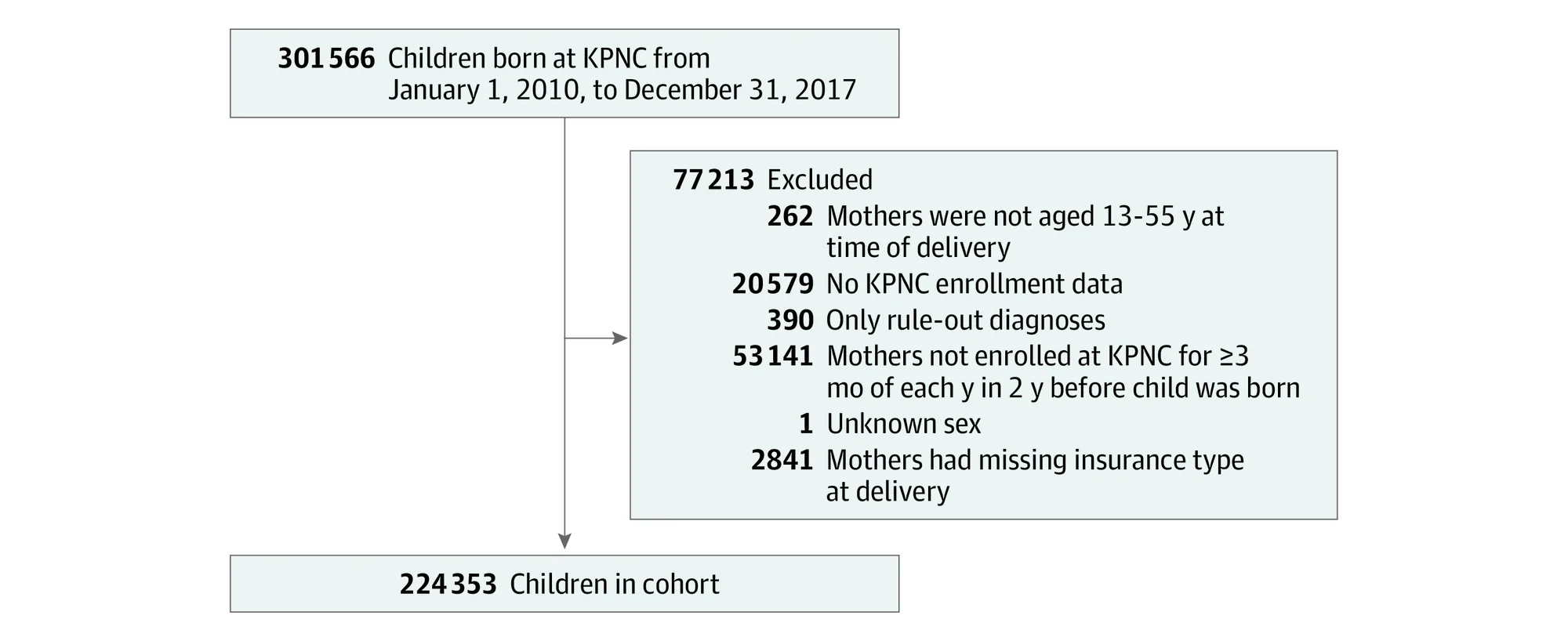
Flowchart Showing Selection of the Study Cohort KPNC indicates Kaiser Permanente Northern California.

### Maternal Diagnoses and Offspring Autism

Of the 42 maternal diagnoses previously identified as associated with autism (eTable 1 in Supplement 1), 25 occurred in at least 5 mothers of children with autism and at least 5 mothers of children without autism in each race and ethnicity group, allowing their inclusion in the analyses (with the exception of other, as described in the Methods section). Among mothers in the study cohort, the median (IQR) number of conditions was 2 (1-3); 93 972 (53.4%) had 2 or more conditions, and 18 846 (10.7%) had 5 or more conditions. The prevalence of each of these diagnoses in the sample, stratified by maternal race and ethnicity and child’s autism status, is presented in eTable 4 in Supplement 1.

Figures 2 and 3 present the associations between each maternal diagnosis and autism, including an interaction term between maternal diagnosis and maternal race and ethnicity. Of the 25 diagnoses analyzed, 4 had a statistically significant interaction term with maternal race and ethnicity, suggesting potential heterogeneity of these associations across maternal race and ethnicity groups. These diagnoses included overweight or obesity, depressive episode, other maternal disease complicating pregnancy, and asthma. Pain associated with female genital organs and menstrual cycle and bipolar disorder were not significant after multiple testing correction (Figure 3). While the point estimates were often highest in White and other race and ethnicity groups, no consistent pattern in the associations was observed across other groups.

**Figure 2.**
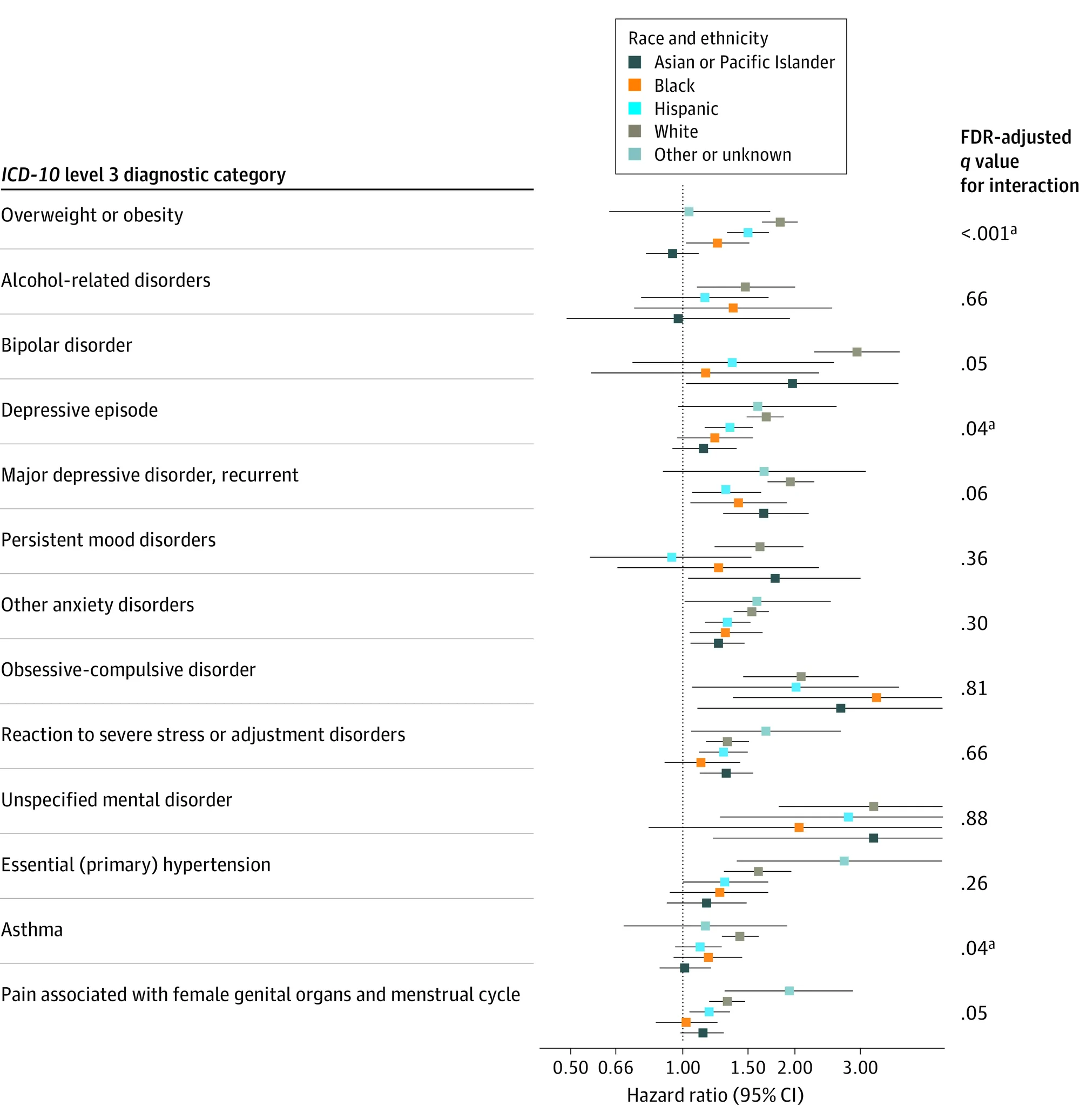
Dot Plot Showing Associations Between *International Statistical Classification of Diseases and Related Health Problems, Tenth Revision (ICD-10*) Level 3 Maternal Diagnoses and Offspring Autism by Race and Ethnicity in the Kaiser Permanente Northern California Cohort ^a^Statistically significant after the false discovery rate (FDR) correction for multiple testing. FDR indicates false discovery rate.

**Figure 3.**
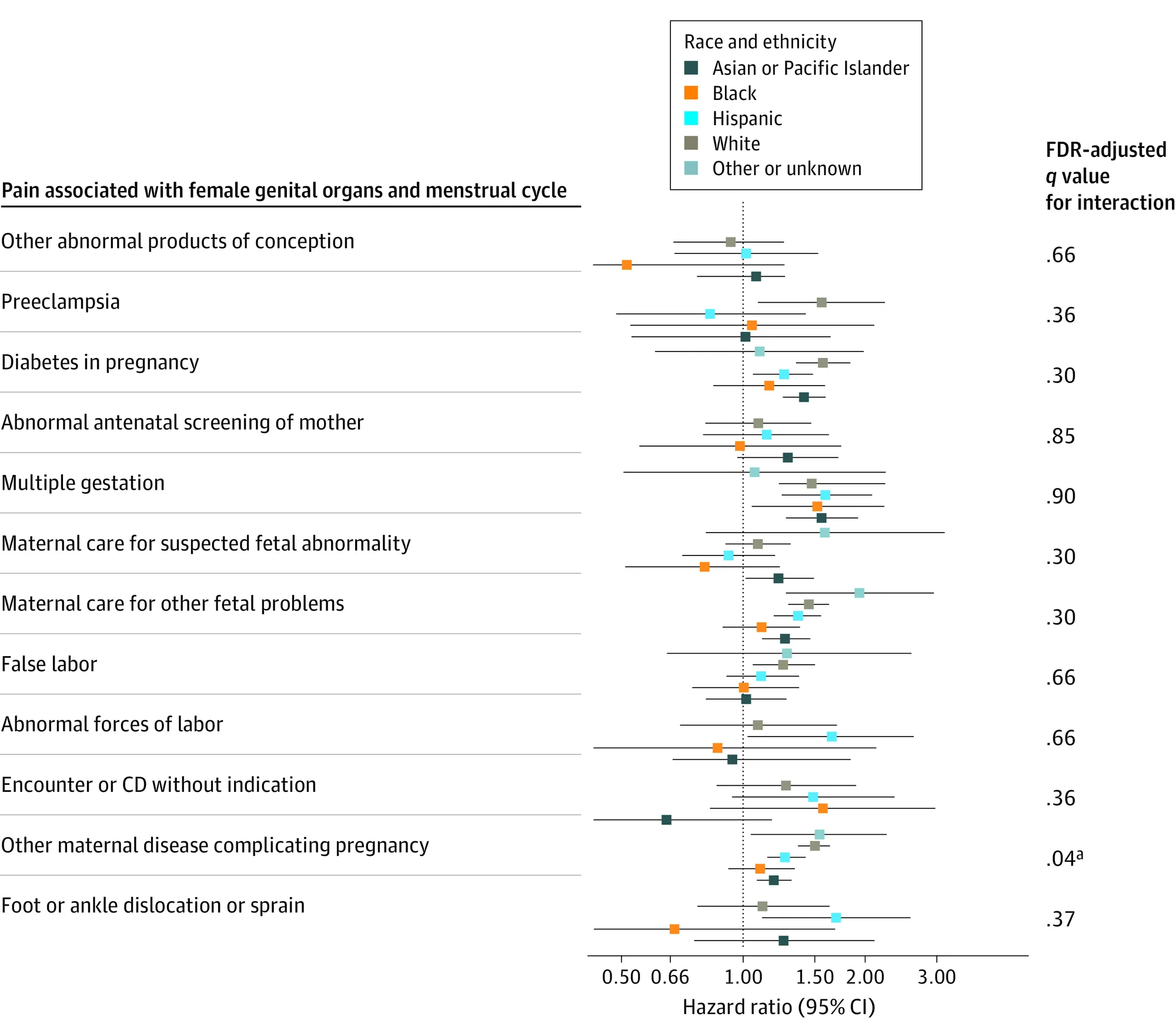
Dot Plot Showing Associations Between *International Statistical Classification of Diseases and Related Health Problems, Tenth Revision* Level 3 Female Genital Organs and Menstrual Cycle Pain Diagnoses and Offspring Autism by Race and Ethnicity in the Kaiser Permanente Northern California Cohort ^a^Statistically significant after the false discovery rate (FDR) correction for multiple testing. CD indicates cesarean delivery.

In the models performed separately in each racial and ethnic group, 10 of the 25 maternal diagnoses were associated with autism in at least 1 group—in every case, the White subgroup—overweight or obesity, depressive episode, major depressive disorder (recurrent), other anxiety disorders, asthma, bipolar disorder, obsessive-compulsive disorder, diabetes, maternal care for other fetal problems, and other maternal disease complicating pregnancy (eTable 6 and the eFigure in Supplement 1). The confidence intervals of most of these associations overlapped across maternal race and ethnicity groups, with 1 exception; for overweight and obesity, the estimate of association was higher in White mothers compared with Black or Asian and Pacific Islander mothers. The stepwise addition of covariates did not result in any substantial differences in the point estimates of the associations between maternal diagnosis and child’s autism.

## Discussion

Overall, the associations between maternal health conditions and offspring autism appeared broadly consistent across maternal racial and ethnic groups. However, for a number of maternal conditions, including obesity, other maternal disease complicating pregnancy, asthma, and depressive episode, these associations differed across maternal race and ethnicity groups. While these differences were not always in a consistent direction, the point estimates tended to be higher in White mothers compared with those in mothers reporting other races and ethnicities, including Black, Hispanic, and Asian and Pacific Islander.

The reasons for these differences are likely complex and heterogeneous across different maternal conditions, warranting further exploration of these associations to identify specific mechanisms. Potential mechanisms underlying the observed differences include the manner in which these maternal conditions are diagnosed, with overdiagnosis or underdiagnosis potentially leading to differential symptom severity in those classified as exposed. For example, a clinical label of obesity is more likely to be given to White vs Black individuals at the same BMI and comorbidity level,^18^ with underdiagnosis of obesity in ethnic minority individuals potentially influencing its weaker observed association with autism in our study. Similar mechanisms could explain lower point estimates associated with depression and bipolar disorder (nominally significant) in children of Black and Hispanic mothers.^19,20^

Additionally, differential causes of certain conditions could also explain the differences in the associations between these maternal conditions and autism. For example, a diagnosis of asthma is more likely to be underlain by environmental factors like air pollution, poorer housing stock, and inadequate ventilation and maternal occupational exposures in Black and Hispanic families compared with White families^21^—potentially introducing differences in the confounder structure across racial and ethnic groups.

Finally, the relevant exposure may be not the diagnosis itself, but how it is managed.^22^ Differences across racial and ethnic groups in access to care, insurance continuity, clinical follow-up, and treatment patterns may affect both disease control and the observed associations in our study. These influences may help explain the heterogeneous associations we observed by reflecting both the potential teratogenic effects of some pregnancy medications and the protective effects of appropriate management of pregnancy complications. This, together with the mechanisms we described previously could render the same observable exposure nonequivalent across diverse racial and ethnic groups.^1,2^

The racial and ethnic differences observed in some associations between maternal health and child autism may, beyond the heterogeneity of factors correlated with maternal diagnoses, reflect disparities in autism diagnosis itself. Historically, autism has been underidentified in racial and ethnic minority children: children of Black mothers were less likely to receive a diagnosis even when scoring above the SRS threshold,^23^ and when minority children were identified, diagnosis tended to occur later, reflecting barriers to assessment services, fewer specialist referrals, evaluation delays, and practitioner bias.^1,24^ Consistent with later and more severe-at-detection identification, minority children were also more likely to have co-occurring diagnoses such as intellectual disability, with milder presentations going unrecognized.^1^ More recently, this identification gap has narrowed, with Black and Hispanic children diagnosed at rates approaching or exceeding those of White children,^25^ a pattern also observed in the current study. Importantly, this narrowing reflects improved case ascertainment, but not necessarily equitable access to screening. Because racial and ethnic minority children in our sample were more likely to be diagnosed with autism, we could not identify a clear mechanism by which differential autism diagnostic rates alone would explain the pattern of associations observed.

### Strengths and Limitations

The strengths of the study include the use of a large, racially and ethnically diverse cohort, detailed EHR data available in KPNC records, robust statistical modeling, and an approach that acknowledges the heterogeneity of both the outcome and exposure. Nonetheless, this study has some limitations. While censoring was accounted for with the use of the Cox model, limited follow-up duration did not enable us to capture autism diagnoses made at later ages, which could be clinically and causally distinct.^26^ The stratification of the analysis rendered the analyses in certain racial and ethnic groups underpowered, resulting in nonsignificant associations despite the overall (sample-wide) significant associations. Similarly, the lack of significant interaction effects between maternal diagnosis and race and ethnicity should not be interpreted as a lack of heterogeneity of the effects explored in our study. Instead, group-specific estimates should be leveraged for joint meta-analytic efforts, enabling a fuller understanding of how maternal health interacts with broader sociodemographic and clinical factors and how these effects impact epidemiological research in autism.^11^

In the future, similar designs should be implemented across other settings, enabling the derivation of joint, more precise estimates. Similarly, race and ethnicity represents only one of the dimensions underlying potential heterogeneity of the observed associations, with others related to the family’s socioeconomic status or migration history, as well as the child’s clinical factors, including familial history or presence of comorbidities.

## Conclusions

In summary, our study demonstrates that while the majority of maternal health conditions during pregnancy are consistently associated with autism across racial and ethnic groups, certain diagnoses—such as obesity, asthma, depressive episodes, and other maternal disease complicating pregnancy—exhibit a similar direction of association but variability in the magnitude of their associations. These differences may reflect disparities in maternal health care access, diagnostic practices, or treatments, as well as autism heterogeneity, arising due to ascertainment. Leveraging large, diverse cohorts and stratified analyses, future research can elucidate these complex interactions further, informing targeted interventions to reduce disparities in maternal and child health outcomes.

## Notes

Supplement 1. eFigure. Associations Between ICD-10 Level 3 Maternal Diagnoses and Offspring Autism Stratified Race and Ethnicity in KPNC Cohort eTable 1. Maternal Diagnoses ICD-10/9 Used for Exposure Definition eTable 2. Composition of the Infant Cohort Other/Unknown Maternal Race and Ethnicity Category (n = 4,806), Based on VDW Demographic Data eTable 3. Demographic Characteristics of the Analytical Sample (Mother-Child Dyads) by Race and Ethnicity eTable 4. Demographic Characteristics of the Analytical Sample (Mother-Child Dyads) by Race and Ethnicity and by ASD Status eTable 5. Frequency of Maternal Diagnosis by Race and Ethnicity and ASD Status eTable 6. Incremental Adjusted (Models 1-4) Associations Between ICD-10 Level 3 Maternal Diagnoses and Offspring Autism by Race and Ethnicity

Supplement 2. Data Sharing Statement
