# Supplementary material for: Racial and Ethnic Differences in Maternal Health and Autism
Source: JAMA Netw Open. 2026 Oct 5;9(10):e2637501. doi: 10.1001/jamanetworkopen.2026.37501 (PMC13639761; doi:10.1001/jamanetworkopen.2026.37501)
Supplement: Supplementary file 1 [file jamanetwopen-e2637501-s001.pdf]

Supplemental Online Content

Ben Messaoud K, Khachadourian V, Anderson M, Janecka M, Croen LA. Racial and ethnic differences in maternal health and autism. *JAMA Netw Open*. 2026;9(10):e2637501. doi:10.1001/jamanetworkopen.2026.37501

eFigure. Associations Between ICD-10 Level 3 Maternal Diagnoses and Offspring Autism Stratified Race and Ethnicity in KPNC Cohort
eTable 1. Maternal Diagnoses ICD-10/9 Used for Exposure Definition
eTable 2. Composition of the Infant Cohort Other/Unknown Maternal Race and Ethnicity Category (n = 4,806), Based on VDW Demographic Data
eTable 3. Demographic Characteristics of the Analytical Sample (Mother-Child Dyads) by Race and Ethnicity
eTable 4. Demographic Characteristics of the Analytical Sample (Mother-Child Dyads) by Race and Ethnicity and by ASD Status
eTable 5. Frequency of Maternal Diagnosis by Race and Ethnicity and ASD Status
eTable 6. Incremental Adjusted (Models 1-4) Associations Between ICD-10 Level 3 Maternal Diagnoses and Offspring Autism by Race and Ethnicity

This supplemental material has been provided by the authors to give readers additional information about their work.

© 2026 [First author last name and initials or group name] et al. [Journal name].

eFigure. Associations Between *ICD-10* Level 3 Maternal Diagnoses and Offspring Autism Stratified Race and Ethnicity in KPNC Cohort

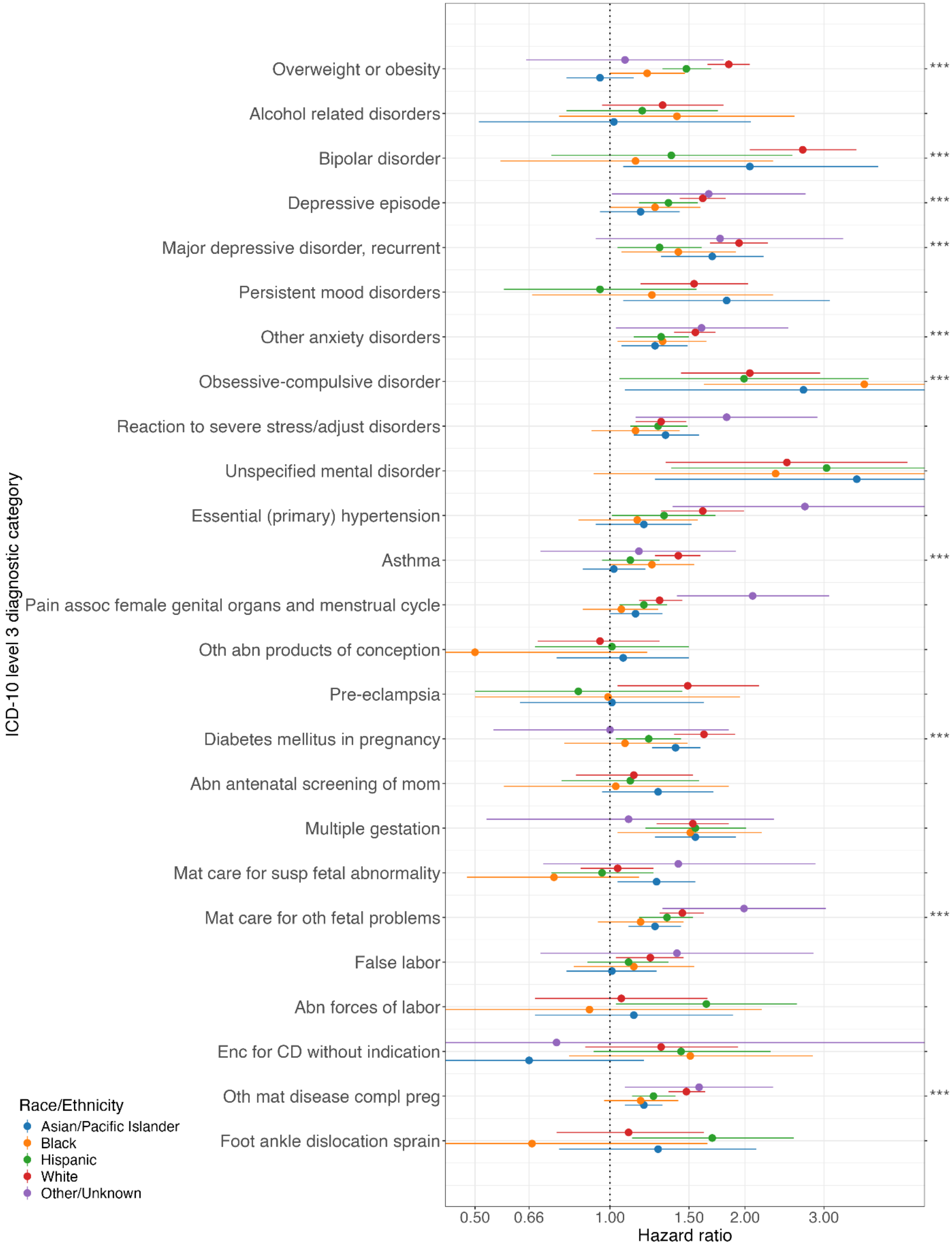

© 2026 [First author last name and initials or group name] et al. [Journal name].

Other/Unknown was a heterogeneous KPNC EHR category that included individuals recorded as Hispanic, Asian, Black/African American, Native Hawaiian/Pacific Islander, American Indian/Alaska Native, White, Multiple Races with Particular Unknown, or Other/Unknown based on other reporting methods.

eTable 1. Maternal Diagnoses *ICD-10/9* Used for Exposure Definition

Diagnosis	ICD-10 code	ICD-9 code
Other abnormal products of conception	O02	631
Pre-eclampsia	O14	642
Diabetes mellitus	O24	250
Abnormal findings on antenatal screening of mother	O28	796
Multiple gestation	O30	651
Maternal care for known or suspected fetal abnormality and damage	O35	655
Maternal care for other fetal problems	O36	656
Premature rupture of membranes	O42	658
False labor	O47	644
Abnormalities of forces of labor	O62	661
Labor and delivery complicated by abnormality of fetal acid-base balance	O68	656
Postpartum hemorrhage	O72	666
Encounter for cesarean delivery without indication	O82	669
Other maternal diseases classifiable elsewhere but complicating pregnancy, childbirth and the puerperium	O99	648
Fracture of skull and facial bones	S02	803
Injury of eye and orbit	S05	918
Dislocation and sprain of joints and ligaments at ankle, foot and toe level	S93	845
Injury of unspecified body region	T14	959
Poisoning by, adverse effect of and underdosing of psychotropic drugs, not elsewhere classified	T43	969
Outcome of delivery	Z37	V27
Malignant neoplasm of breast	C50	174
Overweight or obesity	E66	278
Alcohol related disorders	F10	291, 303, 305
Schizophrenia	F20	295
Schizotypal disorder	F21	301
Bipolar disorder	F31	296
Depressive episode	F32	296
Major depressive disorder, recurrent	F33	296
Persistent mood [affective] disorders	F34	296
Other anxiety disorders	F41	300
Obsessive-compulsive disorder	F42	301
Reaction to severe stress, and adjustment disorders	F43	308
Eating disorders	F50	307
Specific personality disorders	F60	301
Unspecified mental disorder	F99	300
Epilepsy and recurrent seizures	G40	780
Essential (primary) hypertension	I10	401
Pulmonary embolism	I26	415
Asthma	J45	493
Disorder of patella	M22	717
Other systemic involvement of connective tissue	M35	710
Pain and other conditions associated with female genital organs and menstrual cycle	N94	625
Overweight or obesity	E66	278

© 2026 [First author last name and initials or group name] et al. [Journal name].

eTable 2. Composition of the Infant Cohort Other/Unknown Maternal Race and Ethnicity Category (n = 4,806), Based on VDW Demographic Data

VDW-derived race/ethnicity	% of Other/Unknown group	n
Other/Unknown	31.84%	1,530
White	22.04%	1,059
Asian	14.42%	693
Hispanic	13.42%	645
American Indian/Alaska Native	8.34%	401
Multiple Races with Particular Unknown	5.80%	279
Black/African American	3.52%	169
Native Hawaiian/Pacific Islander	0.62%	30
**Total**	**100.00%**	**4,806**

VDW and Infant Cohort race/ethnicity data are derived from different sources and collection methods (survey/self-report vs. health-system–assigned categories) and are not directly comparable; these figures are presented to characterize the heterogeneity of the Other/Unknown category, not to reclassify individuals.

© 2026 [First author last name and initials or group name] et al. [Journal name].

eTable 3. Demographic Characteristics of the Analytical Sample (Mother-Child Dyads) by Race and Ethnicity

Variables	Asian/Pacific Islander 58,993 (26.3%)	Black 15,497 (6.9%)	Hispanic 52,481 (23.4%)	White 92,576 (41.3%)	Other/Unknown 4,806 (2.1%)
**Maternal age at delivery, mean (SD)**	32.5 (4.6)	29.1 (6.4)	29.6 (5.9)	31.5 (5.1)	31.5 (5.3)
**Child ASD status %**	2.9%	3.2%	2.5%	2.0%	2.4%
**Child's sex, n (%)**					
Female	28,719 (48.7%)	7,575 (48.9%)	25,868 (49.3%)	45,117 (48.7%)	2,369 (49.3%)
Male	30,274 (51.3%)	7,922 (51.1%)	26,613 (50.7%)	47,459 (51.3%)	2,437 (50.7%)
**Child's year of birth, n (%)**					
2010	6,353 (10.8%)	1,932 (12.5%)	6,249 (11.9%)	10,563 (11.4%)	669 (13.9%)
2011	6,592 (11.2%)	1,909 (12.3%)	6,172 (11.8%)	10,768 (11.6%)	587 (12.2%)
2012	7,113 (12.0%)	1,894 (12.2%)	6,192 (11.8%)	11,141 (12.0%)	475 (9.9%)
2013	7,009 (11.9%)	1,860 (12.0%)	6,070 (11.6%)	11,373 (12.3%)	518 (10.8%)
2014	7,378 (12.5%)	1,875 (12.1%)	6,461 (12.3%)	11,710 (12.6%)	561 (11.7%)
2015	7,792 (13.2%)	1,931 (12.5%)	6,756 (12.9%)	12,015 (13.0%)	615 (12.8%)
2016	8,273 (14.0%)	1,991 (12.8%)	7,263 (13.8%)	12,425 (13.4%)	663 (13.8%)
2017	8,483 (14.4%)	2,105 (13.6%)	7,318 (13.9%)	12,581 (13.6%)	718 (14.9%)
**Maternal number of days with health care visits the year leading up to delivery, n (%)***					
0-12	17,664 (29.9%)	2,763 (17.8%)	13,207 (25.2%)	22,931 (24.8%)	1,270 (26.4%)
13-16	17,237 (29.2%)	3,684 (23.8%)	14,882 (28.3%)	27,271 (29.4%)	1,341 (27.9%)
17-21	11,210 (19.0%)	3,668 (23.7%)	11,483 (21.9%)	19,986 (21.6%)	985 (20.5%)
22+	12,882 (21.8%)	5,382 (34.7%)	12,909 (24.6%)	22,388 (24.2%)	1,210 (25.2%)
**Insurance Type**					
Government	4,289 (27.7%)	4,289 (27.7%)	6,940 (13.2%)	6,164 (6.7%)	574 (11.4%)
KPNC	11,208 (72.3%)	11,208 (72.3%)	45,541 (86.8%)	86,412 (93.3%)	4,259 (88.6%)

Other/Unknown was a heterogeneous KPNC EHR category that included individuals recorded as Hispanic, Asian, Black/African American, Native Hawaiian/Pacific Islander, American Indian/Alaska Native, White, Multiple Races with Particular Unknown, or Other/Unknown based on other reporting methods.

© 2026 [First author last name and initials or group name] et al. [Journal name].

eTable 4. Demographic Characteristics of the Analytical Sample (Mother-Child Dyads) by Race and Ethnicity and by ASD Status

Race/ethnicity	Asian/Pacific Islander		Black		Hispanic		White		Other &Unknown	
	ASD Diagnosis (n=1,693)	No ASD diagnosis (n=57,300)	ASD Diagnosis (n=493)	No ASD diagnosis (n=15,004)	ASD Diagnosis (n=1,309)	No ASD diagnosis (n=51,172)	ASD Diagnosis (n=1,838)	No ASD diagnosis (n=90,738)	ASD Diagnosis (n=115)	No ASD diagnosis (n=4,691)
**Maternal age at delivery, mean (SD)**	32.5 (4.6)	33.0 (4.7)	31.3 (6.3)	29.0 (6.4)	30.6 (6.0)	29.6 (5.9)	31.6 (5.6)	31.5 (5.1)	33.1 (5.6)	31.5 (5.3)
**Child's sex, n (%)**										
Female	341 (20.1%)	28,378 (49.5%)	98 (19.9%)	7,477 (49.8%)	271 (20.7%)	25,597 (50.0%)	373 (20.3%)	44,744 (49.3%)	20 (17.4%)	2,349 (50.1%)
Male	1,352 (79.9%)	28,922 (50.5%)	395 (80.1%)	7,527 (50.2%)	1,038 (79.3%)	25,575 (50.0%)	1,465 (79.7%)	45,994 (50.7%)	95 (82.6%)	2,342 (49.9%)
**Child's year of birth, n (%)**										
2010	166 (9.8%)	6,187 (10.8%)	66 (13.4%)	1,866 (12.4%)	124 (9.5%)	6,125 (12.0%)	246 (13.4%)	10,317 (11.4%)	18 (15.6%)	651 (13.9%)
2011	179 (10.6%)	6,413 (11.2%)	45 (9.1%)	1,864 (12.4%)	137 (10.5%)	6,035 (11.8%)	250 (13.6%)	10,518 (11.6%)	17 (14.8%)	570 (12.2%)
2012	199 (11.7%)	6,914 (12.1%)	70 (14.2%)	1,824 (12.2%)	160 (12.2%)	6,032 (11.8%)	265 (14.4%)	10,876 (12.0%)	9 (7.8%)	577 (9.9%)
2013	240 (14.2%)	6,769 (11.8%)	77 (15.6%)	1,783 (11.9%)	178 (13.6%)	5,892 (11.5%)	235 (12.8%)	11,138 (12.3%)	11 (9.6%)	507 (10.8%)
2014	242 (14.3%)	7,136 (12.4%)	69 (14.0%)	1,806 (12.0%)	197 (15.0%)	6,264 (12.2%)	266 (14.5%)	11,444 (12.6%)	11 (9.6%)	550 (11.7%)
2015	269 (15.9%)	7,523 (13.1%)	69 (14.0%)	1,862 (12.4%)	212 (16.2%)	6,544 (12.8%)	224 (12.2%)	11,791 (13.0%)	24 (20.9%)	591 (12.6%)
2016	252 (14.9%)	8,021 (96.9%)	55 (11.2%)	1,936 (12.9%)	188 (14.4%)	7,075 (13.8%)	228 (12.4%)	12,197 (13.4%)	11 (9.6%)	652 (13.9%)
2017	146 (8.6%)	8,337 (14.6%)	42 (8.5%)	2,063 (13.8%)	113 (8.6%)	7,205 (14.1%)	124 (6.7%)	12,457 (13.7%)	14 (12.2%)	704 (15.0%)
**Maternal number of days with health care visits the year leading up to delivery, n (%)***										
0-12	416 (24.6%)	17,248 (30.1%)	77 (15.6%)	2,686 (17.9%)	281 (21.5%)	12,926 (25.3%)	316 (17.2%)	22,615 (24.9%)	22 (19.1%)	1,248 (26.6%)
13-16	462 (27.3%)	16,775 (29.3%)	101 (20.5%)	3,583 (23.9%)	302 (23.1%)	14,580 (28.5%)	445 (24.2%)	26,826 (29.6%)	25 (21.7%)	1,316 (28.0%)
17-21	340 (20.1%)	10,870 (19.0%)	120 (24.3%)	3,548 (23.6%)	271 (20.7%)	11,212 (21.9%)	375 (20.4%)	19,611 (21.6%)	20 (17.4%)	965 (20.6%)
22+	475 (28.0%)	12,407 (21.6%)	195 (39.6%)	5,187 (34.6%)	455 (34.7%)	12,454 (24.3%)	702 (38.2%)	21,686 (23.9%)	48 (41.7%)	1,162 (24.8%)
**Insurance Type**										
Government	60 (3.5%)	2,687 (4.7%)	110 (22.3%)	4,179 (27.8%)	188 (14.4%)	6,752 (13.2%)	184 (10.0%)	5,980 (6.6%)	10 (8.7%)	537 (11.4%)
KPNC	1,633 (96.5%)	54,613 (95.3%)	383 (77.7%)	10,825 (72.2%)	1,121 (85.6%)	44,420 (86.8%)	1,654 (90.0%)	84,758 (93.4%)	105 (91.3%)	4,154 (88.6%)

Other/Unknown was a heterogeneous KPNC EHR category that included individuals recorded as Hispanic, Asian, Black/African American, Native Hawaiian/Pacific Islander, American Indian/Alaska Native, White, Multiple Races with Particular Unknown, or Other/Unknown based on other reporting methods.

© 2026 [First author last name and initials or group name] et al. [Journal name].

eTable 5. Frequency of Maternal Diagnosis by Race and Ethnicity and ASD Status

ICD10	Dx description	Asian		Black		Hispanic		White		Other/unknown	
		ASD (N = 1,693)	No ASD (N = 57,300)	ASD (N = 493)	No ASD (N = 15,004)	ASD (N = 1,309)	No ASD (N = 51,172)	ASD (N=1,838)	No ASD (N = 90,738)	ASD (N = 115)	No ASD (N = 4,691)
O02	Other abnormal products of conception	36 (2.1%)	1,088 (1.9%)	5 (1.0%)	301 (2.0%)	25 (1.9%)	988 (1.9%)	45 (2.4%)	2,251 (2.5%)	4 (3.5%)	102 (2.2%)
O14	Pre-eclampsia	17 (1.0%)	533 (0.9%)	10 (2.0%)	268 (1.8%)	14 (1.1%)	601 (1.2%)	37 (2.0%)	1,106 (1.2%)	4 (3.5%)	58 (1.2%)
O24	Diabetes in pregnancy, childbirth, and the puerperium	345 (20%)	8,540 (15%)	45 (9.1%)	1,091 (7.3%)	176 (13%)	5,386 (11%)	189 (10%)	5,877 (6.5%)	12 (10%)	470 (10%)
O28	Abnormal findings on antenatal screening of mother	51 (3.0%)	1,364 (2.4%)	12 (2.4%)	370 (2.5%)	32 (2.4%)	1,134 (2.2%)	44 (2.4%)	2,083 (2.3%)	2 (1.7%)	128 (2.7%)
O30	Multiple gestation	131 (7.7%)	2,888 (5.0%)	44 (8.9%)	862 (5.7%)	89 (6.8%)	2,403 (4.7%)	166 (9.0%)	5,481 (6.0%)	9 (7.8%)	302 (6.4%)
O35	Maternal care for known or suspected fetal abnormality and damage	118 (7.0%)	2,879 (5.0%)	21 (4.3%)	682 (4.5%)	59 (4.5%)	2,212 (4.3%)	124 (6.7%)	4,949 (5.5%)	11 (9.6%)	266 (5.7%)
O36	Maternal care for other fetal problems	257 (15%)	7,420 (13%)	104 (21%)	2,953 (20%)	272 (21%)	8,790 (17%)	368 (20%)	13,917 (15%)	31 (27%)	741 (16%)
O42	Premature rupture of membranes	5 (0.3%)	270 (0.5%)	4 (0.8%)	168 (1.1%)	15 (1.1%)	402 (0.8%)	16 (0.9%)	550 (0.6%)	1 (0.9%)	24 (0.5%)
O47	False labor	80 (4.7%)	2,928 (5.1%)	52 (11%)	1,846 (12%)	105 (8.0%)	4,327 (8.5%)	144 (7.8%)	6,467 (7.1%)	9 (7.8%)	330 (7.0%)
O62	Abnormalities of forces of labor	15 (0.9%)	452 (0.8%)	5 (1.0%)	208 (1.4%)	22 (1.7%)	550 (1.1%)	20 (1.1%)	906 (1.0%)	0 (0%)	35 (0.7%)
O68	Labor and delivery complicated by abnormal fetal acid-base balance	5 (0.3%)	120 (0.2%)	2 (0.4%)	41 (0.3%)	8 (0.6%)	170 (0.3%)	8 (0.4%)	285 (0.3%)	0 (0%)	12 (0.3%)
O72	Postpartum hemorrhage	2 (0.1%)	113 (0.2%)	0 (0%)	26 (0.2%)	5 (0.4%)	122 (0.2%)	6 (0.3%)	223 (0.2%)	0 (0%)	13 (0.3%)
O82	Encounter for cesarean delivery without indication	11 (0.6%)	532 (0.9%)	10 (2.0%)	190 (1.3%)	21 (1.6%)	534 (1.0%)	25 (1.4%)	959 (1.1%)	1 (0.9%)	52 (1.1%)
O99	Other maternal diseases classified elsewhere but complicating pregnancy /childbirth	882 (52%)	27,381 (48%)	324 (66%)	9,873 (66%)	777 (59%)	27,960 (55%)	1,024 (56%)	41,804 (46%)	69 (60%)	2,376 (51%)
S02	Fracture of skull and facial bones	3 (0.2%)	22 (<0.1%)	0 (0%)	42 (0.3%)	1 (<0.1%)	44 (<0.1%)	4 (0.2%)	68 (<0.1%)	1 (0.9%)	4 (<0.1%)
S05	Injury of eye and orbit	4 (0.2%)	109 (0.2%)	2 (0.4%)	55 (0.4%)	4 (0.3%)	95 (0.2%)	7 (0.4%)	241 (0.3%)	0 (0%)	8 (0.2%)
S93	Dislocation and sprain of joints and ligaments at ankle, foot, and toe level	15 (0.9%)	374 (0.7%)	5 (1.0%)	204 (1.4%)	23 (1.8%)	553 (1.1%)	27 (1.5%)	1,144 (1.3%)	3 (2.6%)	51 (1.1%)
T14	Injury of unspecified body region	7 (0.4%)	274 (0.5%)	4 (0.8%)	186 (1.2%)	6 (0.5%)	391 (0.8%)	16 (0.9%)	565 (0.6%)	2 (1.7%)	34 (0.7%)
T43	Psychotropic drugs, not elsewhere classified	1 (<0.1%)	27 (<0.1%)	0 (0%)	22 (0.1%)	0 (0%)	28 (<0.1%)	3 (0.2%)	80 (<0.1%)	0 (0%)	6 (0.1%)
Z37	Outcome of delivery	7 (0.4%)	271 (0.5%)	5 (1.0%)	142 (0.9%)	19 (1.5%)	365 (0.7%)	19 (1.0%)	373 (0.4%)	0 (0%)	24 (0.5%)
C50	Malignant neoplasm of breast	3 (0.2%)	47 (<0.1%)	0 (0%)	5 (<0.1%)	0 (0%)	26 (<0.1%)	1 (<0.1%)	50 (<0.1%)	0 (0%)	2 (<0.1%)
E66	Overweight and obesity	152 (9.0%)	5,533 (9.7%)	173 (35%)	4,485 (30%)	403 (31%)	12,259 (24%)	494 (27%)	15,360 (17%)	18 (16%)	742 (16%)
F10	Alcohol-related disorders	8 (0.5%)	305 (0.5%)	14 (2.8%)	422 (2.8%)	26 (2.0%)	970 (1.9%)	46 (2.5%)	1,724 (1.9%)	3 (2.6%)	57 (1.2%)
F20	Schizophrenia	1 (<0.1%)	10 (<0.1%)	0 (0%)	32 (0.2%)	0 (0%)	22 (<0.1%)	1 (<0.1%)	22 (<0.1%)	0 (0%)	3 (<0.1%)
F21	Schizotypal disorder	0 (0%)	0 (0%)	0 (0%)	0 (0%)	0 (0%)	0 (0%)	0 (0%)	5 (<0.1%)	0 (0%)	0 (0%)
F31	Bipolar disorder	11 (0.6%)	165 (0.3%)	8 (1.6%)	259 (1.7%)	12 (0.9%)	359 (0.7%)	65 (3.5%)	1,229 (1.4%)	3 (2.6%)	52 (1.1%)
F32	Major depressive disorder, single episode	104 (6.1%)	3,126 (5.5%)	99 (20%)	2,712 (18%)	207 (16%)	6,574 (13%)	367 (20%)	11,782 (13%)	20 (17%)	530 (11%)
F33	Major depressive disorder, recurrent	63 (3.7%)	1,279 (2.2%)	55 (11%)	1,290 (8.6%)	97 (7.4%)	3,166 (6.2%)	232 (13%)	6,375 (7.0%)	11 (9.6%)	277 (5.9%)
F34	Persistent mood [affective] disorders	15 (0.9%)	318 (0.6%)	11 (2.2%)	271 (1.8%)	18 (1.4%)	740 (1.4%)	58 (3.2%)	1,784 (2.0%)	4 (3.5%)	71 (1.5%)
F41	Other anxiety disorders	157 (9.3%)	4,318 (7.5%)	99 (20%)	2,531 (17%)	265 (20%)	8,371 (16%)	493 (27%)	17,865 (20%)	26 (23%)	760 (16%)
F42	Obsessive-compulsive disorder	7 (0.4%)	92 (0.2%)	7 (1.4%)	51 (0.3%)	10 (0.8%)	186 (0.4%)	33 (1.8%)	744 (0.8%)	2 (1.7%)	34 (0.7%)
F43	Reaction to severe stress, and adjustment disorders	157 (9.3%)	4,080 (7.1%)	101 (20%)	2,854 (19%)	227 (17%)	7,390 (14%)	294 (16%)	11,504 (13%)	23 (20%)	628 (13%)
F50	Eating disorders	3 (0.2%)	127 (0.2%)	3 (0.6%)	92 (0.6%)	10 (0.8%)	306 (0.6%)	32 (1.7%)	807 (0.9%)	0 (0%)	35 (0.7%)
F60	Specific personality disorders	9 (0.5%)	88 (0.2%)	4 (0.8%)	131 (0.9%)	11 (0.8%)	237 (0.5%)	42 (2.3%)	579 (0.6%)	2 (1.7%)	27 (0.6%)
F99	Mental disorder, not otherwise specified	4 (0.2%)	56 (<0.1%)	5 (1.0%)	84 (0.6%)	8 (0.6%)	137 (0.3%)	13 (0.7%)	194 (0.2%)	0 (0%)	8 (0.2%)
G40	Epilepsy and recurrent seizures	7 (0.4%)	131 (0.2%)	4 (0.8%)	170 (1.1%)	16 (1.2%)	293 (0.6%)	18 (1.0%)	577 (0.6%)	2 (1.7%)	25 (0.5%)
I10	Essential (primary) hypertension	72 (4.3%)	1,862 (3.2%)	51 (10%)	1,128 (7.5%)	62 (4.7%)	1,638 (3.2%)	102 (5.5%)	3,006 (3.3%)	9 (7.8%)	122 (2.6%)
I26	Pulmonary embolism	5 (0.3%)	34 (<0.1%)	1 (0.2%)	27 (0.2%)	1 (<0.1%)	30 (<0.1%)	1 (<0.1%)	92 (0.1%)	0 (0%)	1 (<0.1%)
J45	Asthma	177 (10%)	5,624 (9.8%)	125 (25%)	3,488 (23%)	233 (18%)	8,138 (16%)	400 (22%)	14,686 (16%)	18 (16%)	640 (14%)
M22	Disorder of patella	5 (0.3%)	192 (0.3%)	0 (0%)	101 (0.7%)	3 (0.2%)	214 (0.4%)	13 (0.7%)	474 (0.5%)	0 (0%)	20 (0.4%)
M35	Other systemic involvement of connective tissue	3 (0.2%)	150 (0.3%)	2 (0.4%)	43 (0.3%)	0 (0%)	110 (0.2%)	5 (0.3%)	243 (0.3%)	0 (0%)	14 (0.3%)

© 2026 [First author last name and initials or group name] et al. [Journal name].

ICD10	Dx description	Asian		Black		Hispanic		White		Other/unknown	
		ASD (N = 1,693)	No ASD (N = 57,300)	ASD (N = 493)	No ASD (N = 15,004)	ASD (N = 1,309)	No ASD (N = 51,172)	ASD (N=1,838)	No ASD (N = 90,738)	ASD (N = 115)	No ASD (N = 4,691)
N94	Pain and other conditions associated with female genital organs and menstrual cycle	261 (15%)	7,851 (14%)	164 (33%)	4,864 (32%)	365 (28%)	12,441 (24%)	457 (25%)	17,923 (20%)	40 (35%)	1,043 (22%)

Other/Unknown was a heterogeneous KPNC EHR category that included individuals recorded as Hispanic, Asian, Black/African American, Native Hawaiian/Pacific Islander, American Indian/Alaska Native, White, Multiple Races with Particular Unknown, or Other/Unknown based on other reporting methods.

© 2026 [First author last name and initials or group name] et al. [Journal name].

eTable 6. Incremental Adjusted (Models 1-4) Associations Between *ICD-10* Level 3 Maternal Diagnoses and Offspring Autism by Race and Ethnicity

Model 1: Adjusted for child sex and birth year

Maternal Diagnosis	Asian/Pacific n=58,993				Black n=15,497				Hispanic n=52,481				White n=92,576				Other/Unknown n=4,806			
	HR	95% CI	P Value	Q Value	HR	95% CI	P Value	Q Value	HR	95% CI	P Value	Q Value	HR	95% CI	P Value	Q Value	HR	95% CI	P Value	Q Value
Overweight or obesity	0.96	0.82, 1.14	0.68	0.78	1.27	1.05, 1.54	0.01	0.08	1.48	1.31, 1.67	<0.001	<0.001	1.91	1.71, 2.12	<0.001	<0.001	1.11	0.67, 1.84	0.69	0.80
Depressive episode	1.19	0.97, 1.46	0.09	0.15	1.33	1.06, 1.67	0.02	0.08	1.40	1.20, 1.63	<0.001	<0.001	1.74	1.55, 1.96	<0.001	<0.001	1.72	1.05, 2.82	0.03	0.07
Major depressive disorder, recurrent	1.74	1.34, 2.26	<0.001	<0.001	1.53	1.15, 2.06	0.004	0.05	1.32	1.07, 1.64	0.010	0.02	2.10	1.81, 2.43	<0.001	<0.001	1.85	1.00, 3.42	0.05	0.09
Other anxiety disorders	1.27	1.07, 1.50	0.006	0.02	1.35	1.08, 1.69	0.010	0.08	1.33	1.16, 1.53	<0.001	<0.001	1.61	1.45, 1.79	<0.001	<0.001	1.66	1.07, 2.60	0.03	0.06
Reaction to severe stress/adjust disorders	1.36	1.15, 1.60	<0.001	0.002	1.18	0.94, 1.47	0.16	0.28	1.31	1.13, 1.52	<0.001	0.002	1.38	1.22, 1.58	<0.001	<0.001	1.80	1.13, 2.87	0.01	0.04
Essential (primary) hypertension	1.23	0.96, 1.58	0.10	0.15	1.28	0.95, 1.74	0.11	0.24	1.34	1.03, 1.74	0.03	0.05	1.60	1.30, 1.98	<0.001	<0.001	3.12	1.63, 5.96	0.001	0.004
Asthma	1.04	0.89, 1.22	0.63	0.78	1.27	1.03, 1.57	0.03	0.09	1.17	1.01, 1.35	0.04	0.07	1.50	1.34, 1.69	<0.001	<0.001	1.18	0.72, 1.95	0.52	0.70
Pain associated with female genital organs and the menstrual cycle	1.15	1.00, 1.31	0.05	0.09	1.08	0.89, 1.31	0.42	0.52	1.22	1.08, 1.38	0.002	0.005	1.37	1.22, 1.53	<0.001	<0.001	2.05	1.38, 3.03	<0.001	0.004
Alcohol related disorders	1.05	0.52, 2.12	0.89	0.93	1.50	0.82, 2.76	0.19	0.31	1.34	0.90, 1.98	0.15	0.21	1.68	1.24, 2.27	0.001	0.001	NA	NA	NA	NA
Bipolar disorder	2.19	1.14, 4.20	0.02	0.05	1.30	0.65, 2.64	0.46	0.55	1.50	0.81, 2.77	0.20	0.26	3.36	2.58, 4.39	<0.001	<0.001	NA	NA	NA	NA
Persistent (affective) mood disorders	1.83	1.08, 3.13	0.03	0.06	1.34	0.73, 2.48	0.34	0.47	0.96	0.59, 1.57	0.88	0.95	1.66	1.26, 2.18	<0.001	<0.001	NA	NA	NA	NA
Obsessive-compulsive disorder	2.65	1.06, 6.65	0.04	0.08	4.01	1.81, 8.91	0.001	0.02	1.92	1.01, 3.62	0.05	0.07	2.12	1.49, 3.03	<0.001	<0.001	NA	NA	NA	NA
Unspecified mental disorder	4.39	1.64, 11.77	0.003	0.01	2.56	1.03, 6.32	0.04	0.12	3.83	1.75, 8.39	0.001	0.003	4.31	2.42, 7.69	<0.001	<0.001	NA	NA	NA	NA
Other abnormal products of conception	1.07	0.77, 1.51	0.68	0.78	0.50	0.21, 1.21	0.13	0.24	1.01	0.68, 1.50	0.97	0.97	0.94	0.69, 1.27	0.67	0.68	NA	NA	NA	NA
Pre-eclampsia	1.02	0.64, 1.64	0.93	0.93	0.99	0.50, 1.95	0.97	0.97	0.88	0.52, 1.50	0.65	0.73	1.55	1.08, 2.22	0.02	0.02	NA	NA	NA	NA
Abnormal antenatal screening findings in the mother	1.28	0.97, 1.71	0.08	0.15	1.03	0.58, 1.83	0.92	0.96	1.13	0.79, 1.60	0.51	0.60	1.18	0.88, 1.59	0.28	0.33	NA	NA	NA	NA
Abnormal forces of labor	1.15	0.69, 1.91	0.59	0.78	0.93	0.38, 2.26	0.87	0.94	1.70	1.07, 2.69	0.03	0.05	1.10	0.71, 1.70	0.68	0.68	NA	NA	NA	NA
Foot ankle dislocation sprain	1.28	0.77, 2.14	0.33	0.46	0.73	0.30, 1.81	0.50	0.57	1.72	1.14, 2.60	0.01	0.02	1.14	0.78, 1.66	0.51	0.56	NA	NA	NA	NA
Diabetes mellitus	1.41	1.25, 1.60	<0.001	<0.001	1.14	0.84, 1.56	0.41	0.52	1.21	1.03, 1.42	0.02	0.05	1.64	1.40, 1.91	<0.001	<0.001	1.10	0.60, 2.01	0.77	0.82
Multiple gestation	1.58	1.28, 1.94	<0.001	<0.001	1.54	1.07, 2.23	0.02	0.09	1.55	1.20, 2.01	0.001	0.003	1.51	1.26, 1.81	<0.001	<0.001	1.21	0.59, 2.51	0.60	0.75
Maternal care for suspected fetal abnormality	1.31	1.08, 1.60	0.006	0.02	0.79	0.51, 1.22	0.29	0.45	0.99	0.77, 1.29	0.97	0.97	1.07	0.89, 1.29	0.47	0.53	1.66	0.83, 3.31	0.15	0.25
Maternal care for other fetal problems	1.27	1.10, 1.45	0.001	0.003	1.20	0.97, 1.50	0.10	0.24	1.36	1.19, 1.57	<0.001	<0.001	1.52	1.36, 1.71	<0.001	<0.001	1.98	1.31, 3.01	0.001	0.006
False labor	1.03	0.82, 1.29	0.83	0.90	1.16	0.86, 1.57	0.34	0.47	1.14	0.93, 1.40	0.21	0.26	1.35	1.14, 1.61	0.001	0.001	1.40	0.70, 2.80	0.34	0.51
Encounter for cesarean delivery without indication	0.68	0.38, 1.23	0.21	0.30	1.64	0.88, 3.05	0.12	0.24	1.48	0.94, 2.32	0.09	0.13	1.28	0.87, 1.90	0.21	0.27	0.86	0.12, 6.33	0.88	0.88
Other maternal disease complicating pregnancy	1.20	1.09, 1.33	<0.001	0.001	1.22	1.01, 1.47	0.04	0.11	1.30	1.16, 1.45	<0.001	<0.001	1.58	1.44, 1.73	<0.001	<0.001	1.63	1.11, 2.37	0.01	0.04

All models adjusted

Abbreviations: CI = Confidence Interval, HR = Hazard Ratio; Other/Unknown was a heterogeneous KPNC EHR category that included individuals recorded as Hispanic, Asian, Black/African American, Native Hawaiian/Pacific Islander, American Indian/Alaska Native, White, Multiple Races with Particular Unknown, or Other/Unknown based on other reporting methods.

© 2026 [First author last name and initials or group name] et al. [Journal name].

Model 2: Adjusted for child sex and birth year and maternal age at delivery

Maternal Diagnosis	Asian/Pacific n=58,993				Black n=15,497				Hispanic n=52,481				White n=92,576				Other/Unknown n=4,806			
	HR	95% CI	P Value	Q Value	HR	95% CI	P Value	Q Value	HR	95% CI	P Value	Q Value	HR	95% CI	P Value	Q Value	HR	95% CI	P Value	Q Value
Overweight or obesity	0.96	0.81, 1.14	0.62	0.78	1.27	1.04, 1.54	0.02	0.08	1.51	1.33, 1.71	<0.001	<0.001	1.90	1.71, 2.12	<0.001	<0.001	1.10	0.67, 1.83	0.71	0.85
Depressive episode	1.17	0.95, 1.43	0.13	0.22	1.33	1.06, 1.67	0.01	0.08	1.39	1.19, 1.61	<0.001	<0.001	1.67	1.48, 1.88	<0.001	<0.001	1.68	1.03, 2.75	0.04	0.08
Major depressive disorder, recurrent	1.70	1.30, 2.21	<0.001	<0.001	1.52	1.13, 2.04	0.005	0.07	1.33	1.07, 1.65	0.009	0.02	2.02	1.74, 2.35	<0.001	<0.001	1.79	0.95, 3.38	0.07	0.13
Other anxiety disorders	1.26	1.07, 1.49	0.007	0.02	1.35	1.08, 1.69	0.010	0.08	1.35	1.17, 1.55	<0.001	<0.001	1.59	1.43, 1.77	<0.001	<0.001	1.62	1.04, 2.54	0.03	0.08
Reaction to severe stress/adjust disorders	1.34	1.13, 1.58	0.001	0.003	1.17	0.94, 1.47	0.16	0.31	1.30	1.12, 1.52	<0.001	0.002	1.34	1.18, 1.53	<0.001	<0.001	1.84	1.15, 2.92	0.01	0.04
Essential (primary) hypertension	1.20	0.93, 1.53	0.16	0.25	1.22	0.90, 1.65	0.21	0.33	1.35	1.04, 1.76	0.03	0.05	1.64	1.33, 2.03	<0.001	<0.001	2.81	1.45, 5.48	0.002	0.01
Asthma	1.02	0.87, 1.20	0.78	0.88	1.29	1.04, 1.59	0.02	0.09	1.15	0.99, 1.33	0.07	0.10	1.45	1.29, 1.63	<0.001	<0.001	1.17	0.71, 1.94	0.53	0.73
Pain associated with female genital organs and the menstrual cycle	1.15	1.00, 1.31	0.05	0.09	1.10	0.91, 1.33	0.34	0.47	1.21	1.07, 1.38	0.002	0.008	1.32	1.18, 1.48	<0.001	<0.001	2.10	1.42, 3.11	<0.001	0.003
Alcohol related disorders	1.04	0.52, 2.09	0.92	0.95	1.49	0.82, 2.73	0.19	0.32	1.24	0.84, 1.83	0.28	0.36	1.41	1.04, 1.92	0.03	0.04	NA	NA	NA	NA
Bipolar disorder	2.09	1.08, 4.02	0.03	0.06	1.32	0.65, 2.67	0.44	0.55	1.46	0.79, 2.72	0.23	0.32	3.09	2.36, 4.05	<0.001	<0.001	NA	NA	NA	NA
Persistent (affective) mood disorders	1.82	1.07, 3.10	0.03	0.06	1.31	0.71, 2.42	0.39	0.51	0.95	0.58, 1.55	0.84	0.88	1.58	1.20, 2.08	0.001	0.002	NA	NA	NA	NA
Obsessive-compulsive disorder	2.69	1.07, 6.75	0.03	0.07	4.02	1.80, 8.97	0.001	0.02	2.00	1.06, 3.78	0.03	0.05	2.08	1.45, 2.97	<0.001	<0.001	NA	NA	NA	NA
Unspecified mental disorder	3.52	1.25, 9.92	0.02	0.05	2.60	1.05, 6.47	0.04	0.11	3.30	1.51, 7.22	0.003	0.008	2.78	1.49, 5.19	0.001	0.002	NA	NA	NA	NA
Other abnormal products of conception	1.07	0.76, 1.50	0.70	0.83	0.50	0.21, 1.20	0.12	0.27	1.01	0.68, 1.51	0.95	0.95	0.95	0.70, 1.30	0.76	0.79	NA	NA	NA	NA
Pre-eclampsia	1.01	0.63, 1.62	0.97	0.97	0.97	0.50, 1.91	0.94	0.94	0.88	0.52, 1.50	0.64	0.73	1.54	1.08, 2.21	0.02	0.03	NA	NA	NA	NA
Abnormal antenatal screening findings in the mother	1.28	0.96, 1.70	0.09	0.16	1.04	0.58, 1.84	0.90	0.94	1.11	0.78, 1.58	0.56	0.66	1.16	0.86, 1.57	0.32	0.38	NA	NA	NA	NA
Abnormal forces of labor	1.14	0.68, 1.89	0.62	0.78	0.93	0.38, 2.27	0.87	0.94	1.68	1.06, 2.68	0.03	0.05	1.06	0.68, 1.65	0.79	0.79	NA	NA	NA	NA
Foot ankle dislocation sprain	1.28	0.77, 2.12	0.35	0.48	0.72	0.29, 1.78	0.48	0.57	1.71	1.13, 2.59	0.01	0.03	1.14	0.78, 1.66	0.51	0.58	NA	NA	NA	NA
Diabetes mellitus	1.41	1.24, 1.59	<0.001	<0.001	1.10	0.80, 1.51	0.54	0.62	1.24	1.05, 1.46	0.01	0.03	1.66	1.42, 1.94	<0.001	<0.001	1.01	0.55, 1.86	0.97	0.97
Multiple gestation	1.55	1.25, 1.91	<0.001	<0.001	1.51	1.04, 2.18	0.03	0.10	1.56	1.21, 2.02	0.001	0.002	1.51	1.25, 1.81	<0.001	<0.001	1.13	0.55, 2.35	0.74	0.85
Maternal care for suspected fetal abnormality	1.27	1.04, 1.55	0.02	0.06	0.74	0.48, 1.15	0.18	0.32	0.97	0.75, 1.27	0.84	0.88	1.04	0.86, 1.26	0.70	0.76	1.43	0.71, 2.87	0.32	0.48
Maternal care for other fetal problems	1.26	1.10, 1.45	0.001	0.005	1.20	0.97, 1.50	0.10	0.24	1.35	1.18, 1.55	<0.001	<0.001	1.48	1.31, 1.66	<0.001	<0.001	2.01	1.32, 3.06	0.001	0.008
False labor	1.02	0.81, 1.28	0.89	0.95	1.18	0.87, 1.60	0.30	0.44	1.12	0.91, 1.37	0.29	0.37	1.27	1.06, 1.51	0.008	0.01	1.43	0.71, 2.89	0.32	0.48
Encounter for cesarean delivery without indication	0.66	0.36, 1.19	0.17	0.25	1.59	0.85, 2.97	0.14	0.30	1.51	0.96, 2.37	0.07	0.11	1.31	0.88, 1.94	0.18	0.23	0.77	0.10, 5.68	0.80	0.85
Other maternal disease complicating pregnancy	1.19	1.08, 1.32	<0.001	0.002	1.23	1.02, 1.48	0.03	0.10	1.28	1.14, 1.43	<0.001	<0.001	1.52	1.38, 1.67	<0.001	<0.001	1.60	1.10, 2.33	0.01	0.04

All models adjusted

Abbreviations: CI = Confidence Interval, HR = Hazard Ratio; Other/Unknown was a heterogeneous KPNC EHR category that included individuals recorded as Hispanic, Asian, Black/African American, Native Hawaiian/Pacific Islander, American Indian/Alaska Native, White, Multiple Races with Particular Unknown, or Other/Unknown based on other reporting methods.

© 2026 [First author last name and initials or group name] et al. [Journal name].

Model 3: Adjusted for child sex and birth year, maternal age and insurance type at delivery

Maternal Diagnosis	Asian/Pacific n=58,993				Black n=15,497				Hispanic n=52,481				White n=92,576				Other/Unknown n=4,806			
	HR	95% CI	P Value	Q Value	HR	95% CI	P Value	Q Value	HR	95% CI	P Value	Q Value	HR	95% CI	P Value	Q Value	HR	95% CI	P Value	Q Value
Overweight or obesity	0.95	0.80, 1.13	0.57	0.75	1.21	1.00, 1.47	0.05	0.18	1.48	1.31, 1.68	<0.001	<0.001	1.84	1.65, 2.05	<0.001	<0.001	1.08	0.65, 1.79	0.76	0.85
Depressive episode	1.17	0.95, 1.43	0.14	0.24	1.26	1.00, 1.59	0.05	0.18	1.35	1.16, 1.57	<0.001	0.001	1.61	1.43, 1.81	<0.001	<0.001	1.66	1.01, 2.73	0.04	0.10
Major depressive disorder, recurrent	1.69	1.30, 2.20	<0.001	0.001	1.42	1.06, 1.91	0.02	0.17	1.29	1.05, 1.60	0.02	0.04	1.94	1.67, 2.25	<0.001	<0.001	1.76	0.93, 3.31	0.08	0.15
Other anxiety disorders	1.26	1.06, 1.49	0.008	0.03	1.31	1.04, 1.64	0.02	0.17	1.30	1.13, 1.50	<0.001	0.001	1.55	1.39, 1.72	<0.001	<0.001	1.60	1.03, 2.50	0.04	0.10
Reaction to severe stress/adjust disorders	1.33	1.13, 1.58	0.001	0.004	1.14	0.91, 1.43	0.26	0.45	1.28	1.11, 1.49	0.001	0.004	1.30	1.14, 1.48	<0.001	<0.001	1.82	1.14, 2.90	0.01	0.05
Essential (primary) hypertension	1.19	0.93, 1.53	0.16	0.24	1.15	0.85, 1.57	0.36	0.57	1.32	1.01, 1.72	0.04	0.07	1.61	1.30, 1.99	<0.001	<0.001	2.72	1.38, 5.36	0.004	0.02
Asthma	1.02	0.87, 1.20	0.79	0.90	1.24	1.00, 1.54	0.05	0.18	1.11	0.96, 1.29	0.16	0.23	1.42	1.26, 1.59	<0.001	<0.001	1.16	0.70, 1.91	0.57	0.77
Pain associated with female genital organs and the menstrual cycle	1.14	1.00, 1.31	0.05	0.10	1.06	0.87, 1.28	0.56	0.70	1.19	1.05, 1.35	0.008	0.02	1.29	1.16, 1.45	<0.001	<0.001	2.08	1.41, 3.08	<0.001	0.004
Alcohol related disorders	1.02	0.51, 2.06	0.95	0.97	1.41	0.77, 2.58	0.27	0.45	1.18	0.80, 1.74	0.41	0.51	1.31	0.96, 1.79	0.08	0.11	NA	NA	NA	NA
Bipolar disorder	2.05	1.07, 3.96	0.03	0.07	1.14	0.57, 2.31	0.71	0.81	1.37	0.74, 2.55	0.32	0.44	2.69	2.05, 3.54	<0.001	<0.001	NA	NA	NA	NA
Persistent (affective) mood disorders	1.82	1.07, 3.09	0.03	0.07	1.24	0.67, 2.31	0.49	0.64	0.95	0.58, 1.56	0.84	0.88	1.54	1.17, 2.03	0.002	0.003	NA	NA	NA	NA
Obsessive-compulsive disorder	2.69	1.08, 6.73	0.03	0.07	3.69	1.63, 8.37	0.002	0.05	1.99	1.05, 3.77	0.03	0.06	2.06	1.44, 2.94	<0.001	<0.001	NA	NA	NA	NA
Unspecified mental disorder	3.55	1.27, 9.94	0.02	0.05	2.34	0.92, 5.95	0.07	0.23	3.04	1.37, 6.76	0.006	0.02	2.48	1.33, 4.61	0.004	0.006	NA	NA	NA	NA
Other abnormal products of conception	1.07	0.76, 1.50	0.69	0.82	0.50	0.21, 1.21	0.12	0.31	1.01	0.68, 1.50	0.97	0.97	0.95	0.70, 1.29	0.73	0.76	NA	NA	NA	NA
Pre-eclampsia	1.01	0.63, 1.63	0.97	0.97	0.99	0.50, 1.95	0.98	0.98	0.85	0.50, 1.45	0.56	0.63	1.50	1.04, 2.15	0.03	0.04	NA	NA	NA	NA
Abnormal antenatal screening findings in the mother	1.28	0.96, 1.70	0.09	0.16	1.04	0.58, 1.84	0.90	0.94	1.11	0.78, 1.58	0.56	0.63	1.16	0.86, 1.57	0.32	0.38	NA	NA	NA	NA
Abnormal forces of labor	1.14	0.68, 1.89	0.62	0.78	0.93	0.38, 2.27	0.87	0.94	1.68	1.06, 2.68	0.03	0.05	1.06	0.68, 1.65	0.79	0.79	NA	NA	NA	NA
Foot ankle dislocation sprain	1.28	0.77, 2.12	0.35	0.48	0.72	0.29, 1.78	0.48	0.64	1.71	1.13, 2.59	0.01	0.03	1.14	0.78, 1.66	0.51	0.58	NA	NA	NA	NA
Diabetes mellitus	1.41	1.24, 1.59	<0.001	<0.001	1.08	0.79, 1.49	0.62	0.74	1.22	1.03, 1.44	0.02	0.04	1.62	1.39, 1.90	<0.001	<0.001	1.00	0.55, 1.84	0.99	0.99
Multiple gestation	1.55	1.26, 1.91	<0.001	0.001	1.51	1.04, 2.18	0.03	0.18	1.55	1.20, 2.01	0.001	0.004	1.53	1.27, 1.84	<0.001	<0.001	1.10	0.53, 2.32	0.79	0.85
Maternal care for suspected fetal abnormality	1.27	1.04, 1.55	0.02	0.05	0.75	0.48, 1.16	0.20	0.38	0.96	0.74, 1.25	0.78	0.85	1.04	0.86, 1.25	0.71	0.76	1.42	0.71, 2.87	0.32	0.50
Maternal care for other fetal problems	1.26	1.10, 1.44	0.001	0.005	1.18	0.94, 1.46	0.15	0.34	1.34	1.16, 1.54	<0.001	0.001	1.45	1.29, 1.62	<0.001	<0.001	1.99	1.31, 3.03	0.001	0.009
False labor	1.01	0.80, 1.27	0.93	0.97	1.13	0.83, 1.54	0.44	0.64	1.10	0.89, 1.35	0.37	0.49	1.23	1.03, 1.46	0.02	0.03	1.41	0.70, 2.84	0.33	0.50
Encounter for cesarean delivery without indication	0.66	0.36, 1.19	0.17	0.24	1.51	0.81, 2.83	0.19	0.38	1.45	0.92, 2.28	0.11	0.18	1.30	0.88, 1.93	0.19	0.23	0.76	0.10, 5.60	0.79	0.85
Other maternal disease complicating pregnancy	1.19	1.08, 1.31	<0.001	0.003	1.17	0.97, 1.42	0.10	0.28	1.25	1.12, 1.40	<0.001	0.004	1.48	1.35, 1.63	<0.001	<0.001	1.58	1.08, 2.31	0.02	0.05

All models adjusted

Abbreviations: CI = Confidence Interval, HR = Hazard Ratio; Other/Unknown was a heterogeneous KPNC EHR category that included individuals recorded as Hispanic, Asian, Black/African American, Native Hawaiian/Pacific Islander, American Indian/Alaska Native, White, Multiple Races with Particular Unknown, or Other/Unknown based on other reporting methods.

© 2026 [First author last name and initials or group name] et al. [Journal name].

Model 4: Adjusted for child sex and birth year, maternal age and insurance type at delivery, and number of days in the year prior to delivery in which mom had an encounter

Maternal Diagnosis	Asian/Pacific n=58,993				Black n=15,497				Hispanic n=52,481				White n=92,576				Other/Unknown n=4,806			
	HR	95% CI	P Value	Q Value	HR	95% CI	P Value	Q Value	HR	95% CI	P Value	Q Value	HR	95% CI	P Value	Q Value	HR	95% CI	P Value	Q Value
Overweight or obesity	0.86	0.73, 1.03	0.09	0.21	1.17	0.96, 1.43	0.11	0.37	1.37	1.21, 1.56	<0.001	<0.001	1.64	1.47, 1.83	<0.001	<0.001	0.87	0.52, 1.46	0.60	0.76
Depressive episode	1.06	0.87, 1.31	0.55	0.77	1.20	0.94, 1.52	0.14	0.37	1.21	1.03, 1.41	0.02	0.13	1.39	1.23, 1.57	<0.001	<0.001	1.32	0.78, 2.24	0.29	0.54
Major depressive disorder, recurrent	1.52	1.16, 1.98	0.002	0.03	1.34	0.99, 1.82	0.06	0.37	1.12	0.90, 1.40	0.30	0.50	1.60	1.36, 1.87	<0.001	<0.001	1.37	0.73, 2.57	0.33	0.54
Other anxiety disorders	1.15	0.97, 1.37	0.10	0.21	1.24	0.97, 1.58	0.08	0.37	1.17	1.02, 1.35	0.03	0.13	1.34	1.19, 1.50	<0.001	<0.001	1.30	0.82, 2.08	0.27	0.54
Reaction to severe stress/adjust disorders	1.24	1.04, 1.47	0.02	0.10	1.08	0.85, 1.37	0.52	0.81	1.17	1.00, 1.36	0.05	0.15	1.12	0.98, 1.28	0.10	0.17	1.56	0.97, 2.53	0.07	0.35
Essential (primary) hypertension	1.05	0.82, 1.34	0.72	0.82	1.08	0.79, 1.47	0.62	0.81	1.13	0.86, 1.47	0.39	0.57	1.27	1.02, 1.59	0.03	0.07	1.87	0.90, 3.89	0.10	0.36
Asthma	0.96	0.82, 1.13	0.66	0.82	1.20	0.96, 1.49	0.11	0.37	1.03	0.89, 1.19	0.72	0.89	1.27	1.13, 1.43	<0.001	<0.001	1.01	0.61, 1.69	0.95	0.95
Pain associated with female genital organs and the menstrual cycle	1.07	0.94, 1.23	0.31	0.49	1.01	0.82, 1.23	0.95	0.99	1.09	0.96, 1.24	0.19	0.36	1.14	1.02, 1.28	0.02	0.06	1.81	1.20, 2.73	0.005	0.07
Alcohol related disorders	0.95	0.47, 1.90	0.88	0.92	1.35	0.73, 2.48	0.33	0.56	1.05	0.71, 1.56	0.80	0.89	1.03	0.75, 1.41	0.86	0.86	NA	NA	NA	NA
Bipolar disorder	1.71	0.88, 3.31	0.11	0.22	1.03	0.50, 2.11	0.93	0.99	1.06	0.56, 2.00	0.86	0.89	1.99	1.50, 2.64	<0.001	<0.001	NA	NA	NA	NA
Persistent (affective) mood disorders	1.67	0.98, 2.85	0.06	0.16	1.18	0.63, 2.19	0.60	0.81	0.84	0.51, 1.38	0.50	0.69	1.31	0.99, 1.73	0.06	0.11	NA	NA	NA	NA
Obsessive-compulsive disorder	2.37	0.95, 5.93	0.06	0.16	3.33	1.42, 7.81	0.006	0.14	1.61	0.85, 3.05	0.15	0.32	1.71	1.19, 2.46	0.004	0.010	NA	NA	NA	NA
Unspecified mental disorder	3.25	1.15, 9.21	0.03	0.13	2.08	0.79, 5.46	0.14	0.37	2.45	1.09, 5.54	0.03	0.13	1.49	0.80, 2.77	0.20	0.30	NA	NA	NA	NA
Other abnormal products of conception	0.99	0.71, 1.39	0.95	0.95	0.47	0.20, 1.14	0.10	0.37	0.91	0.61, 1.36	0.65	0.86	0.84	0.62, 1.14	0.26	0.34	NA	NA	NA	NA
Pre-eclampsia	0.91	0.56, 1.47	0.70	0.82	0.95	0.49, 1.87	0.89	0.99	0.75	0.44, 1.29	0.30	0.50	1.29	0.90, 1.86	0.17	0.26	NA	NA	NA	NA
Abnormal antenatal screening findings in the mother	1.21	0.91, 1.61	0.18	0.31	1.01	0.57, 1.80	0.98	0.99	1.03	0.73, 1.48	0.85	0.89	1.06	0.79, 1.43	0.70	0.77	NA	NA	NA	NA
Abnormal forces of labor	1.05	0.63, 1.74	0.85	0.92	0.81	0.33, 2.00	0.65	0.81	1.48	0.93, 2.35	0.10	0.25	0.92	0.59, 1.43	0.71	0.77	NA	NA	NA	NA
Foot ankle dislocation sprain	1.19	0.71, 1.97	0.51	0.75	0.63	0.25, 1.56	0.32	0.56	1.53	1.01, 2.33	0.04	0.15	0.96	0.65, 1.40	0.82	0.86	NA	NA	NA	NA
Diabetes mellitus	1.26	1.11, 1.44	0.001	0.01	1.00	0.72, 1.38	0.99	0.99	1.02	0.85, 1.21	0.86	0.89	1.27	1.08, 1.50	0.004	0.010	0.64	0.32, 1.28	0.21	0.52
Multiple gestation	1.34	1.08, 1.67	0.008	0.07	1.43	0.98, 2.08	0.06	0.37	1.36	1.05, 1.76	0.02	0.13	1.22	1.01, 1.48	0.04	0.07	0.81	0.37, 1.79	0.60	0.76
Maternal care for suspected fetal abnormality	1.21	1.00, 1.48	0.06	0.16	0.72	0.46, 1.12	0.15	0.37	0.88	0.68, 1.15	0.36	0.57	0.95	0.79, 1.16	0.64	0.76	1.32	0.66, 2.65	0.43	0.64
Maternal care for other fetal problems	1.17	1.02, 1.34	0.03	0.13	1.12	0.89, 1.41	0.32	0.56	1.21	1.05, 1.39	0.009	0.11	1.25	1.11, 1.41	<0.001	0.001	1.68	1.08, 2.61	0.02	0.17
False labor	0.94	0.75, 1.19	0.63	0.82	1.09	0.80, 1.48	0.60	0.81	1.01	0.82, 1.25	0.91	0.91	1.08	0.90, 1.29	0.42	0.53	1.03	0.47, 2.27	0.94	0.95
Encounter for cesarean delivery without indication	0.63	0.35, 1.14	0.13	0.22	1.51	0.81, 2.82	0.20	0.45	1.39	0.88, 2.19	0.15	0.32	1.26	0.85, 1.88	0.24	0.33	0.71	0.10, 5.16	0.74	0.85
Other maternal disease complicating pregnancy	1.10	1.00, 1.22	0.06	0.16	1.11	0.91, 1.36	0.31	0.56	1.12	0.99, 1.26	0.06	0.18	1.28	1.16, 1.41	<0.001	<0.001	1.29	0.87, 1.91	0.21	0.52

All models adjusted

Abbreviations: CI = Confidence Interval, HR = Hazard Ratio; Other/Unknown was a heterogeneous KPNC EHR category that included individuals recorded as Hispanic, Asian, Black/African American, Native Hawaiian/Pacific Islander, American Indian/Alaska Native, White, Multiple Races with Particular Unknown, or Other/Unknown based on other reporting methods.

© 2026 [First author last name and initials or group name] et al. [Journal name].
