# Supplementary material for: Racial and Ethnic Differences in Maternal Health and Autism
Source: JAMA Netw Open. 2026 Oct 5;9(10):e2637501. doi: 10.1001/jamanetworkopen.2026.37501 (PMC13639761; doi:10.1001/jamanetworkopen.2026.37501)
Supplement: Supplementary file 2 [file jamanetwopen-e2637501-s002.pdf]

Data Sharing Statement

Ben Messaoud. Racial and Ethnic Differences in Maternal Health and Autism. *JAMA Netw Open*. Published October 05, 2026. doi:10.1001/jamanetworkopen.2026.37501

Data
Data available: No
